# Supplementary material for: Demonstration of a superconducting diode-with-memory, operational at zero magnetic field with switchable nonreciprocity
Source: Nat Commun. 2022 Jun 27;13:3658. doi: 10.1038/s41467-022-31256-w (PMC9237109; doi:10.1038/s41467-022-31256-w)
Supplement: Supplementary file 1 — Supplementary Information [file 41467_2022_31256_MOESM1_ESM.pdf]

Supplementary information to the manuscript: Demonstration of a superconducting diode-with-memory, operational at zero magnetic field with switchable non-reciprocity.

I. SAMPLES

Studied devices contain planar JJs. D1 is made from a bi-layer Nb (70 nm, top)/CuNi(50 nm, bottom) with superparamagnetic CuNi. D2 is made from a single Nb (70 nm) film. The films are deposited by dc-magnetron sputtering at room temperature on an oxidised Si wafer. Films are first patterned into $\sim$ 6 μm-wide bridges by photolithography and reactive ion etching, and subsequently nano-patterned by Ga^+ focused ion beam (FIB) FEI Nova 200. Both Nb-CuNi-Nb (D1) and Nb-c-Nb (D2) JJs have a variable-thickness-bridge structure. They are made by cutting a narrow groove in the top Nb layer by FIB. Single line cut, with zero nominal width, is performed at 10 pA current with best focusing (focal spot $\sim$ 10 nm). Etching time is calculated automatically for the chosen nominal depth and volume-per-dose. Presented Nb-CuNi-Nb (D1) and Nb-c-Nb (D2) JJs are made using nominal depths of 70 nm and 100 nm, correspondingly, using volume-per-dose 0.27 μm^3/nC (standard for Si etch). However, as discussed in Ref. [46], the actual depth of the cut is reduced by redeposition of Nb. The groove has a V-shape profile with the aspect ratio (depth/width) $\sim$ 2. From the image in Fig. 1 (a) it can be seen that the groove width at the surface is ($\sim 20-30$ nm), from which the actual depth can be estimated $\sim 40-60$ nm. Self-limiting of the cut depth is consistent with a slow increase of junction resistances with increasing nominal depth [46]. It is also obvious from Nb-c-Nb JJs on D2, which exhibit large I_c despite the nominal depth (100 nm) being larger than the film thickness (70 nm). The vortex trap (a hole $\sim$ 50 nm in diameter) is also made by FIB. We fabricated and tested similar JJs with other metals in the bottom layer [3,42-48]. All of them work in a similar manner and results do not depend neither on the presence or specific material of the bottom layer, nor on the depth of the cut.

Both devices D1,2 have similar cross-like geometry, as can be seen from Fig. 3 (b) and the right panel in Supplementary Figure 1, and have practically identical dimensions. In the left panel of Fig. S1 we show SEM image from Fig. 3 (a) with specified geometrical parameters. Junction lengths, $L \simeq 5.6$ μm (in x-direction), separation between JJs $\simeq 1.35$ μm (in z-direction). The vortex trap is placed in the middle $x_v \simeq L/2 \simeq 2.8$ μm, at a distance $z_{v1} \simeq 0.6$ μm from JJ1 and $z_{v2} \simeq 0.75$ μm from JJ2. Corresponding polar angles are $\Theta_{v1} = 2\arctan(x_v/z_{v1}) \simeq 0.87\,\pi$ and $\Theta_{v2} = 2\arctan(x_v/z_{v2}) \simeq 0.83\,\pi$.

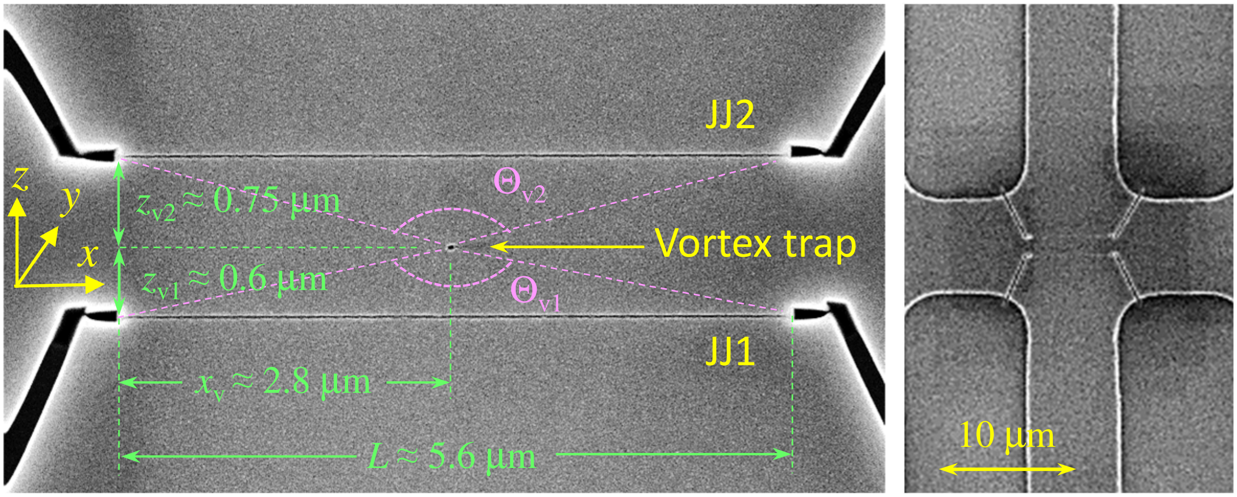

Supplementary Figure 1.

Specification of device geometry. SEM images of the D1 (Nb-CuNi-Nb) device at different magnifications. The left panel shows the same image as in Fig. 3 (a) with specification of geometrical parameters. The right panel demonstrates the cross-like geometry of the device with four electrodes.

Parameters of Nb films.

Pearl length for similar Nb-CuNi-Nb JJs at $T = 6.7$ K was estimated in Ref. [43] as, $\lambda_P \simeq 300$ nm. It was deduced from observation of a crossover to the mesoscopic limit for Abrikosov vortex-iduced Josephson phase shift. Assuming the empirical temperature dependence of the London penetration depth $\lambda(T) = \lambda(0)[1-(T/T_c)^4]^{-1/2}$ and taking $T_c = 8.4$ K, this gives $\lambda(0) = 112$ nm. The in-plain coherence length, $\xi(0) \simeq 14$ nm, was estimated from analysis of the upper critical field in Ref. [50].

II. TEMPERATURE DEPENDENCE

Supplementary Figure 2 shows the resistive transition of JJ1 on D1. The main transition in panel (a) occurs at $T_c(Nb) \simeq 8.4$ K. Here the large resistance ($\sim 30\ \Omega$) originates from mm-long Nb electrodes. At lower T a small shoulder develops in $R(T)$ with $R \sim 0.1\ \Omega$, as shown in Fig. S2 (b). It is approximately equal to the junction resistance. At elevated T, measured resistance is slightly larger than $R_n = 0.065\ \Omega$, deduced from the I-Vs in Fig. 3 (f). This is due to a parasitic flux-flow contribution from Nb electrodes. It appears because of a quasi-four-probe (superconducting two-probe) measurement configuration, which includes a part of electrode resistance in case if it is non-zero.

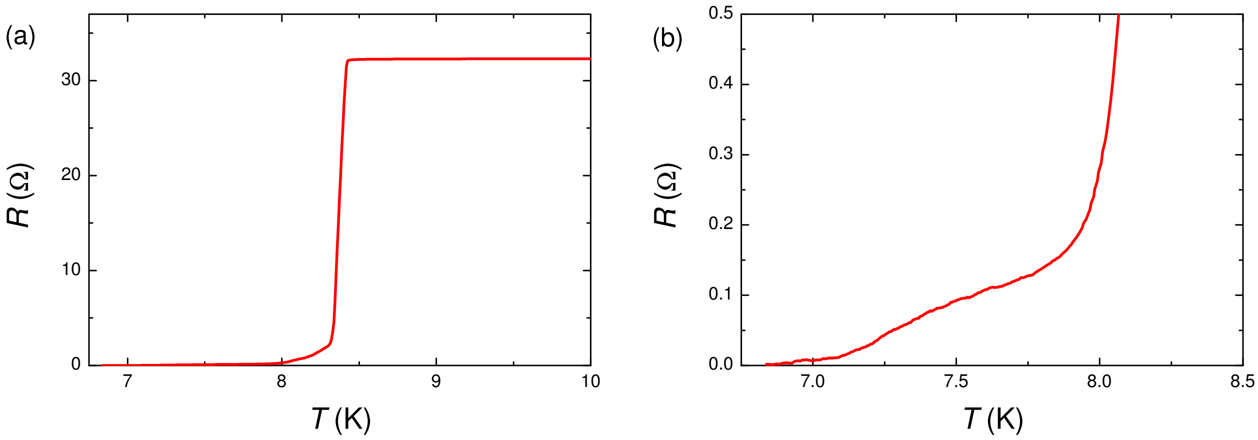

Supplementary Figure 2. **Temperature dependence of resistance for the D1 device.** (a) The resistive transition of junction 1 on D1 (Nb-CuNi-Nb). (b) A close up on the junction transition. Measurements are done at $I_{ac} \simeq 1.5\ \mu$A in ambient field.

Supplementary Fig. 3 (a) shows the I-V characteristics of JJ1 on D2 at different T. Fig. S3 (b) shows the temperature dependence of the critical current. Fig. S4 shows a set of I-Vs in a broader T-range for a similar Nb-c-Nb JJ on another device. Fig. S5 shows $I(H)$ modulations for JJ1 on D2 at different temperatures, measured using left-corner bias and without trapped vortices. Disruptions in the $I_c(H)$ patterns are caused by profound synchronization (current locking) of the two JJs on the device, as discussed in Ref. [45]

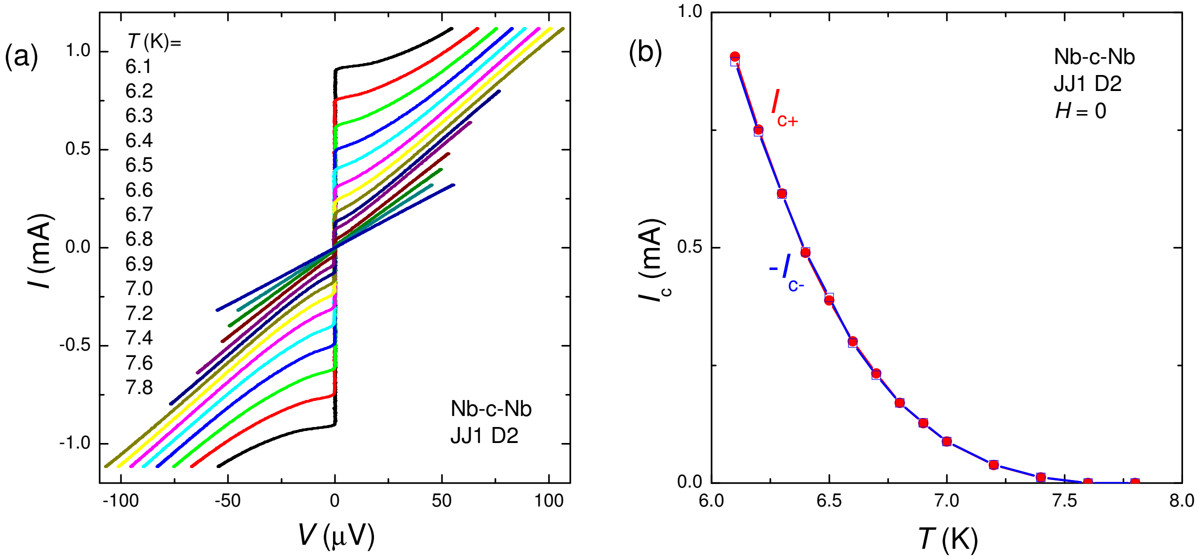

Supplementary Figure 3. **Temperature variation of the current-voltage characteristics for the D2 device.** (a) I-V characteristics of Nb-c-Nb JJ1 on D2 at different temperatures. (b) Temperature dependence of the critical current for JJ1 of D2.

Self-field effect is determined by the effective inductance, L_{sf}, and current, $\delta\Phi_{sf} = L_{sf} I$. L_{sf} depends on geometry and is

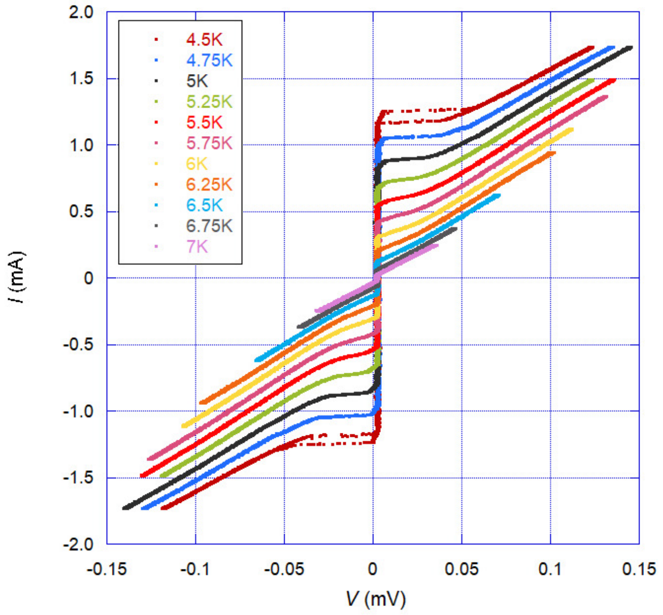

Supplementary Figure 4. **Temperature dependence of the I-V characteristics in a broader T-range.** I-Vs of a Nb-c-Nb JJ on another device. Appearance of a hysteresis at $T \lesssim 4.5$ K can be seen.

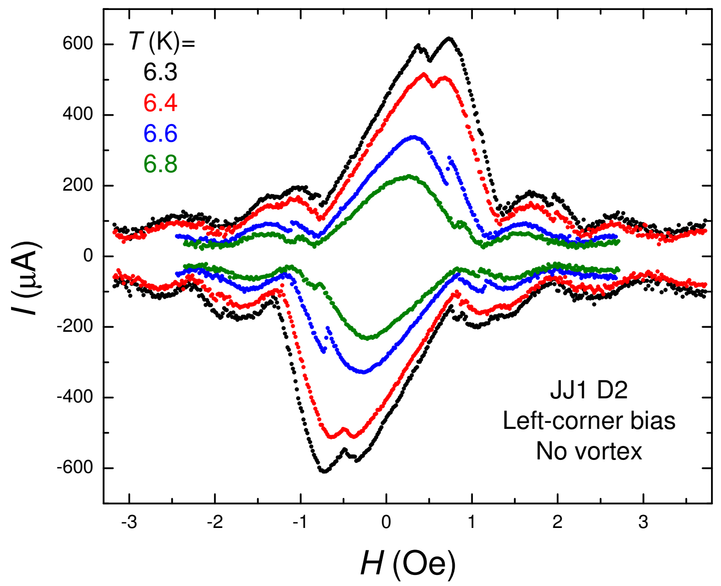

Supplementary Figure 5. **Temperature dependence of the $I_c(H)$ modulation patterns for the D2 device.** $I(H)$ modulations for JJ1 on D2 at different temperatures. Measurements are done using left-corner bias and without trapped vortices.

approximately T-independent. This is seen from the T-independent tilt of the $I_c(H)$ patterns in Fig. S5. On the other hand, the critical current has a strong, superlinear $I_c(T)$ dependence for both types of JJs, as shown in Fig. S3 (b). Therefore, temperature affects the self-field induced flux at the critical current. Due to the strong $I_c(T)$ dependence, $\delta\Phi_{sf}$ rapidly increases with decreasing T, as seen from Fig. S5. However, at low T JJs enter the long junction limit, $L > 4\lambda_J$, which we want to avoid. Furthermore, increased I_c and decreased heat conductivity at low T may cause appearance of hysteresis, which is unwanted for diode operation. As seen from Fig. S4, the hysteresis appears below $\sim$ 4.5 K. This explains the choice of the operation temperature $\sim 6-7$ K in the manuscript. It is adopted in order to keep JJs in the short limit, without hysteresis of I-Vs, but with high enough self-field flux $\delta\Phi_{sf} \sim \Phi_0$.

III. VORTEX MANIPULATION BY CURRENT PULSES

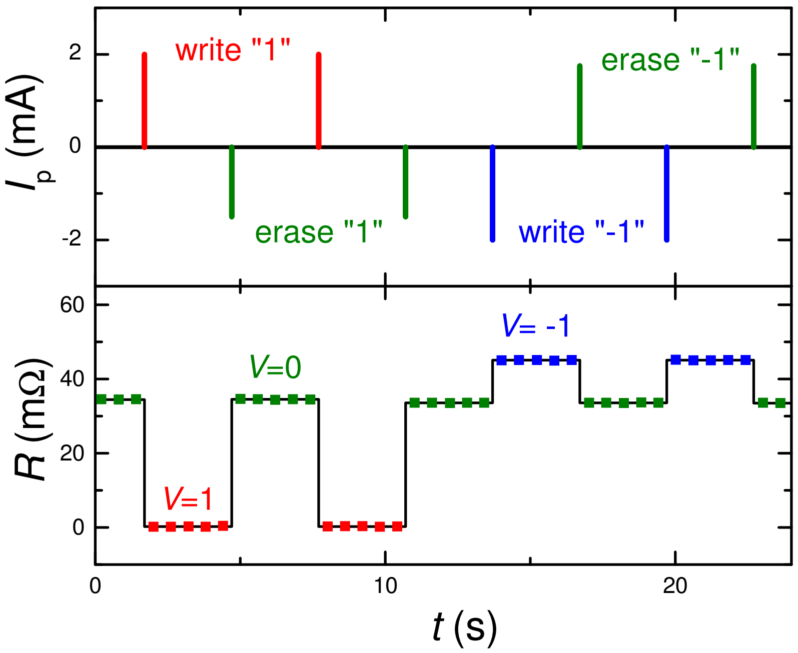

Supplementary Figure 6. **Demonstration of vortex manipulation and readout for Nb-CuNi-Nb sample.** Top panel shows a current pulse train. Red and blue pulses introduce a vortex (write "1") and an antivortex (write "-1"), olive pulses remove (erase) vortices. Bottom panel - the corresponding time dependence of readout junction resistance. Switching between vortex-free, $V = 0$ (olive), vortex, $V = 1$ (red), and antivortex, $V = -1$ (blue symbols), states can be clearly seen. Measurements are done at $T \simeq 6.8$ K and $H \simeq 0.85$ Oe. A finite field was applied solely for allowing a simple distinction between vortex and antivortex states and is not needed for vortex manuipulation.

Vortices are manipulated by short current pulses, as described in Ref. [3]. Supplementary Figure. 5. shows the procedure of trapping (write) and removing (erase) of Abricosov vortices in the trap for a Nb-CuNi-Nb JJ. Vortex states are prepared in the following way. We start from the Meissner state by zero-field cooling of a device without bias current. Vortices are introduced by applying current pulses with, or without external magnetic field. The top panel in Fig. S6 shows an example of pulse train. The shortest, instrumentation limited pulse duration in our system is $\sim 17\ \mu$s. The resulting vorticity, V, in the trap state is established from the analysis of $I_c(H)$ distortion. Once established, it can be comfortably deduced from resistance of a nearby readout JJ. This is demonstrated in the bottom panel of Fig. S6, which shows time dependence of junction resistance for the pulse train from the top panel. This experiment is performed at $H = 0.85$ Oe, close to the flux quantization field, H_0, in order to facilitate a clear distinction between vortex, $V = 1$, and antivortex, $V = -1$, states. Since $H \sim H_0$, the vortex-free $V = 0$ state has lower I_c than the vortex state $V = 1$. Consequently, $R(V = -1) > R(V = 0) > R(V = 1)$. However, external field is not needed for vortex manipulation.

From Fig. S6 it can be seen that, starting from the Meissner state, $V = 0$, we can introduce either a vortex, or an antivortex, by sending a positive or a negative pulse, correspondingly. They are erased by pulses in the reverse directions: negative for a vortex and positive for an antivortex. This indicates that vortices always enter and leave the device from one side, corresponding to the positive x-direction (right side) in the image of Fig. 3 (a). Upon closer inspection of Fig. 3 (a) it is possible to see a narrow gray line going from the vortex trap in this direction. It originates from the beam blanking procedure of the FEI Nova 200 FIB: the beam is not turned off but is just parked outside the field of view. This creates a shallow track and introduces the asymmetry of pinning potential, resulting in the preferential vortex motion at the right side of the trap. Such asymmetry is important for controllable vortex manipulation.

Supplementary Figures 7 and 8 demonstrate vortex manipulation in D2 at zero field. Top panel in Fig. S7 shows pulse trains with linearly increasing positive and negative amplitudes. Switching between "0", "1" and "-1" states occurs at specific pulse amplitudes and leads to abrupt changes of I_c, as shown in the bottom panel of Fig. S7. Fig. S8 demonstrates controllable and reproducible manipulation of vortex states for the D2 device.

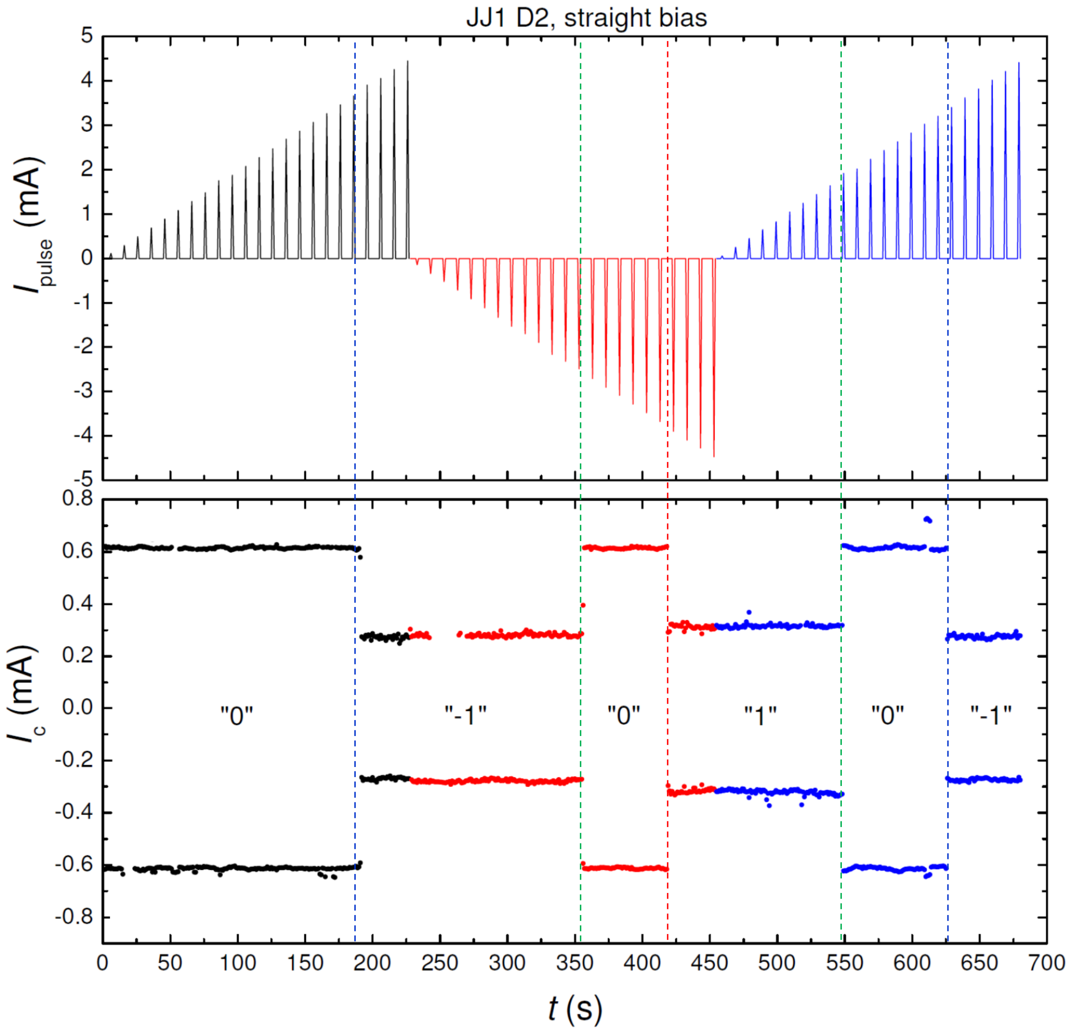

Supplementary Figure 7. **Demonstration of vortex manipulation in D2 (Nb-c-Nb) at zero field.** Top panel shows a current pulse train with linearly increasing positive and negative amplitudes. Bottom panel shows simultaneously measured positive and negative critical currents of JJ1. Sequential switching between “0”, “1” and “-1” vortex states is marked by vertical dashed lines. Measurements are done at $T \simeq 6.3$ K and $H = 0$. Current is applied in the straight bias configuration.

IV. RECTIFICATION OF AC-SIGNALS

Nonreciprocity allows rectification of the ac-signal, as demonstrated in Fig. 5 of the main manuscript. As seen from the bottom panel in Fig. 5 (a), for $I_{ac} < I_{c+}$ (red, olive and blue curves) only negative voltage develops during each ac-cycle. This leads to rectification of the ac signal with development of negative time-averaged dc-voltage $\langle V \rangle$, as shown in Fig. 5 (b). For $I_{ac} > I_{c+}$, see magenta and black curves in Fig. 5 (a), an additional positive peak appears in $V(t)$, which leads to reduction of the rectified dc voltage $|\langle V \rangle|$, as can be seen from Fig. 5 (b). The green line in Fig. 5 (b) represents the maximum rectified voltage, $|\langle V \rangle_{max}| = I_{ac} R_n / \pi$, for the ideal case of infinite nonreciprocity, represented by the green I-V in Fig. 5 (a). Bottom panel in Fig. 5 (b) shows the efficiency of rectification relative to the ideal case $|\langle V \rangle / \langle V \rangle_{max}|$. At $I_{ac} = I_{c+}$ it reaches the maximum value $\simeq 80\%$, which is close to the value $1 - 1/\nu$, where $\nu = |I_{c-}/I_{c+}|$ is the nonreciprocity.

Rectification is caused by the difference of positive and negative voltages during the ac-bias period. Therefore, the sum of dc-voltages at opposite dc-currents, $\Sigma V = V(I) + V(-I)$, gives a qualitative estimate of rectification. In Supplementary Figure 9 we show magnetic field dependencies of dc-voltages at five dc-currents with both polarities. Left (a) and right (b) panels correspond the cases of Figs. 4 (a) and (c) without vortex and with antivortex, respectively. The sum $\Sigma V = V(I) + V(-I)$ gives a hint about magnetic field and bias dependencies of rectification.

Rectification data presented in Fig. S9 and in the manuscript is obtained at the frequency of $f = 23$ Hz. The frequency range

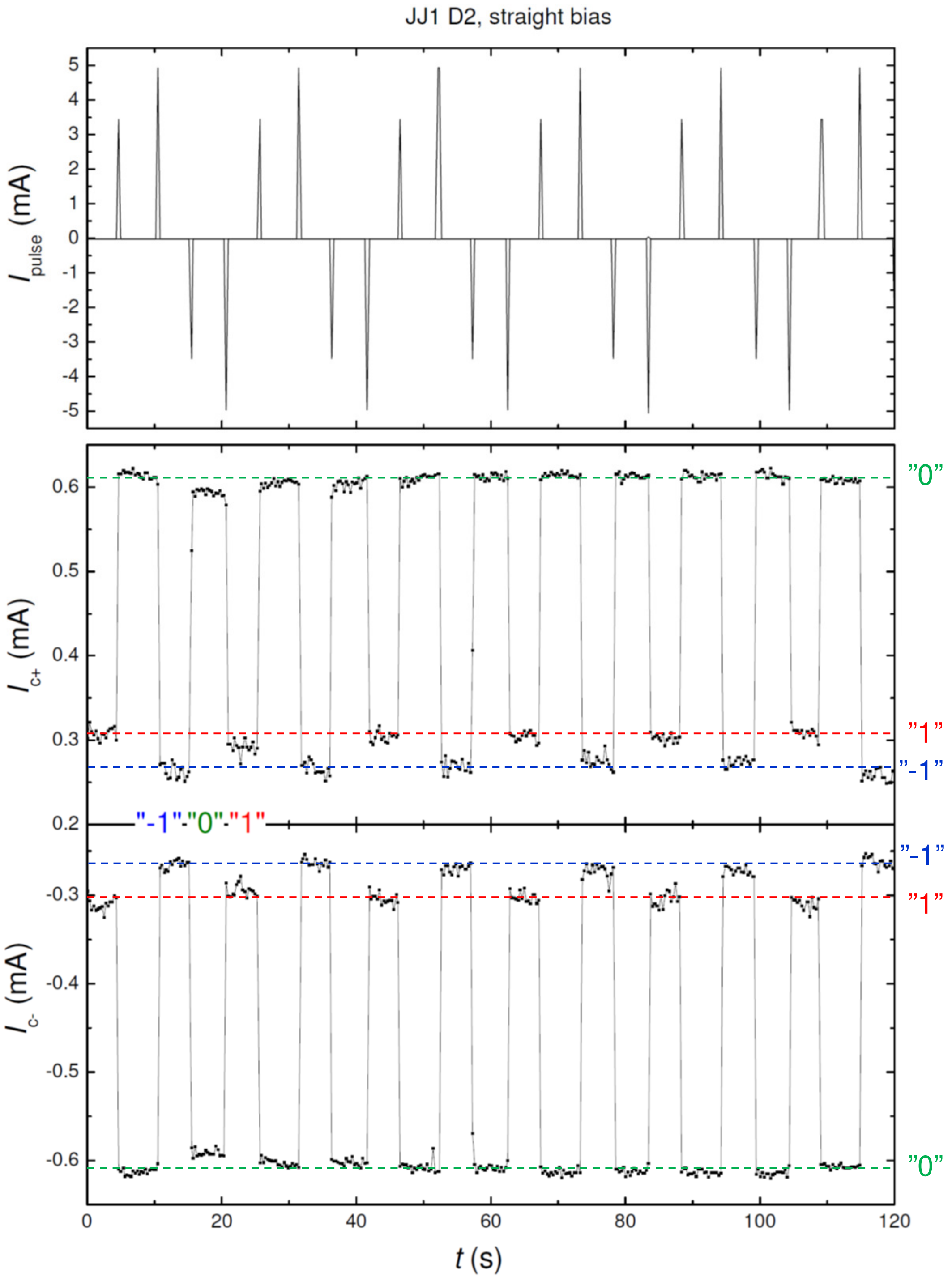

Supplementary Figure 8. **Demonstration of controllable vortex manipulation in D2 at zero field.** Top panel shows a periodic current pulse train. Middle and bottom panels show simultaneously measured time dependence of positive and negative critical currents of JJ1. It is seen that the chosen pulse train leads to a periodic switching "0" - "-1" - "0" -"1". Measurements are done at $T \simeq 6.3$ K and $H = 0$. Current is applied in the straight bias configuration.

in our setup is limited by *RLC* filtering to about a kHz. Fundamentally, the operation frequency range of a JJ is determined by the characteristic frequency, $f_c \sim I_c R_n/\Phi_0$, which can be in excess of 10 GHz for our Nb-CuNi-Nb JJs. For Nb-c-Nb JJs with higher $I_c R_n$, up to $\sim$ 1 mV [45], the frequency can be in the range of several hundred GHz. But, processing of so high frequencies requires a proper microwave design with coaxial cables to the sample and transmission lines at the chip. Such a technique is

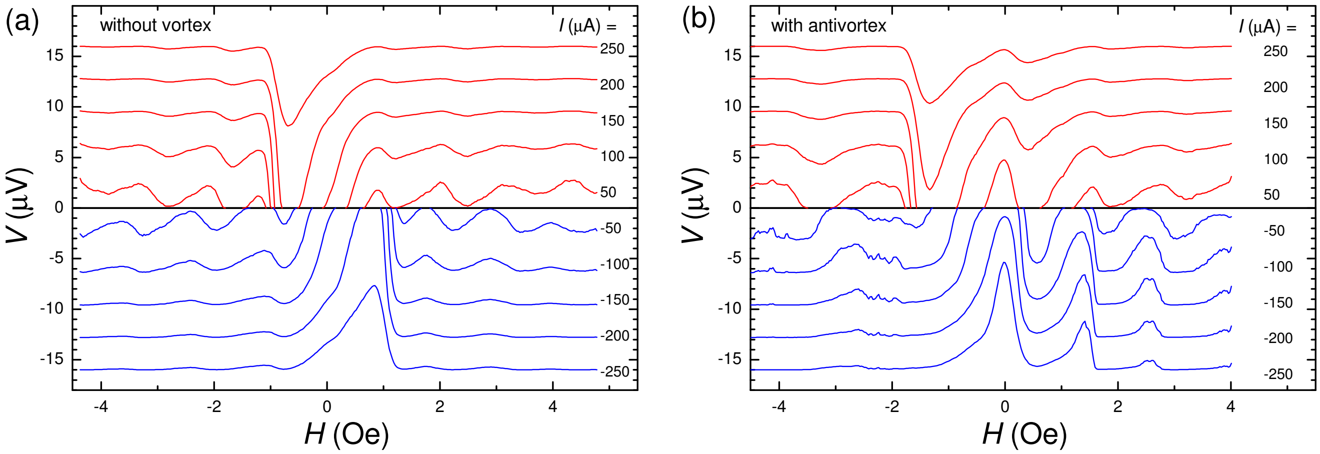

Supplementary Figure 9. **Analysis of magnetic field dependencies of dc-voltages at positive and negative dc-currents.** Left (a) and right (b) panels correspond the cases of Figs. 4 (a) and (c) without vortex and with antivortex, respectively. The sum $\Sigma V = V(I) + V(-I)$ gives a hint about magnetic field and bias dependencies of rectification.

well established and widely used for analysis of microwave properties of Josephson electronics.
